# Taxonomic revision of the genus ***Callimerus*** Gorham ***s. l*.** (Coleoptera, Cleridae). Part I. ***latifrons*** species-group

**DOI:** 10.3897/zookeys.294.4669

**Published:** 2013-04-22

**Authors:** Gan-Yan Yang, Olivier Montreuil, Xing-Ke Yang

**Affiliations:** 1 Key Laboratory of Zoological Systematics and Evolution, Institute of Zoology, Chinese Academy of Sciences, No. 1 Beichen West Road, Chaoyang District, Beijing, 100101, P.R. China; 2 UMR 7205, Département de Systématique et Évolution, Muséum National d’Histoire Naturelle, CP 50, 57 rue Cuvier, F-75231 Paris, Cedex 05, France

**Keywords:** Cleridae, *Callimerus*, *Brachycallimerus*, Oriental region, systematics, new species, synonymy, species group

## Abstract

The *latifrons* species-group (=*Brachycallimerus* *sensu* Chapin 1924, Corporaal 1950; = *flavofasciatus*-group *sensu* Kolibáč 1998) of *Callimerus* Gorham is redefined and revised. Five species are recognized including one new species *Callimerus cacuminis* Yang & Yang **sp. n.** (type locality: Yunnan, China). *Callimerus flavofasciatus* Schenkling, 1902 is newly synonymized with *Callimerus latifrons* Gorham, 1876. *Callimerus trifasciatus* Schenkling, 1899a is transferred to the genus *Corynommadius* Schenkling, 1899a. *Callimerus gorhami* Corporaal, 1949 and *Callimerus pallidus* Gorham, 1892 are excluded from the *latifrons* species-group (their assignment to a species-group will be dealt with in a subsequent paper). A key to species of the *latifrons* species-group is given and habitus of each type specimen, male terminalia, and other diagnostic characters are illustrated.

## Introduction

The genus *Brachycallimerus* Chapin was erected by Chapin (1924) for *Callimerus latifrons* Gorham, 1876, *Callimerus latesignatus* Gorham, 1892, *Callimerus rusticus* Gorham, 1883, *Callimerus pectoralis* Schenkling, 1899b and *Callimerus trifasciatus* Schenkling, 1899a, which differed from typical *Callimerus* Gorham in having “broad and compact form, short and compact antennae, and the total absence of scales from the upper surface”. Later, two additional species, *Brachycallimerus doesburgi* Corporaal, 1937 and *Brachycallimerus gorhami* Corporaal, 1949, were described and another two species, *Callimerus pallidus* Gorham, 1892 and *Callimerus flavofasciatus* Schenkling, 1902, were transferred to *Brachycallimerus* (Corporaal 1937; Chapin 1924). Hence, in the catalogue Corporaal (1950), a total of 9 valid species were included in this genus.

Kolibáč (1998) synonymized *Brachycallimerus* with *Callimerus* based on phylogenetic analysis of 38 morphological characters stating that “*Brachycallimerus* is derived from the major part of *Callimerus*” and that “*Callimerus* would be a paraphyletic taxon if *Brachycallimerus* were classified as a separate genus”. He treated members of *Brachycallimerus* as *flavofasciatus*-group infra *Callimerus*. Other three species groups were proposed within *Callimerus* in that paper: *dulcis*-group (major part of *Callimerus* Gorham, 1876), *coomani*-group (= *Cucujocallimerus* Pic, 1929) and *prasinatus*-group (= *Stenocallimerus* Corporaal & Pic, 1940). *Brachycallimerus doesburgi* Corporaal, 1937 was excluded from *flavofasciatus*-group, but assigned in *coomani*-group for the reason that its claw lacks a basal tooth (Fig. 77). The synonymy of *Brachycallimerus* under *Callimerus* is approved in Gerstmeier et al. (2012: 391), though they were treated as separate genera in Opitz (2010: 82).

In the present paper, we follow the classification system of Kolibáč (1998) and treat *Brachycallimerus* Chapin in a species-group rank, named it *latifrons* species-group (= *flavofasciatus*-group *sensu* Kolibáč 1998); the name of the species group is changed because *Callimerus flavofasciatus* Schenkling, 1902 is synonymized with *Callimerus latifrons* Gorham, 1876 herein, which is the oldest species of this species group. The purpose of this paper is to redefine *latifrons* species-group and revise its members. Five species are kept in the redefined *latifrons* species-group, with one new species from China (Yunnan) and Laos; one new synonym is proposed and three species that formally belong to this species group are excluded. The exclusion of *Callimerus doesburgi* (Corporaal, 1937) by Kolibáč (1998) is approved. All members of this species group are distributed in Southeast Asia.

### Material and methods

Materials examined in the present paper are deposited in the following collections. Abbreviations are shown in the text as follows:

CAUChina Agricultural University, Beijing, China


CCCCCollection of Mr. CHEN Changchin, Taiwan, China


IZASInstitute of Zoology, Chinese Academy of Sciences, Beijing, China


MCSNMuseo Civico di Storia Naturale, Genova, Italy


MNHNMuséum National d’Histoire Naturelle, Paris, France


NHMBNaturhistorisches Museum, Basel, Switzerland


NHMLThe Natural History Museum, London, United Kingdom


NHRSNaturhistoriska Riksmuseet, Stockholm, Sweden


OUMHope Department of Entomology, University Museum, Oxford, United Kingdom


RMNHRijksmuseum van Natuurlijke Historie, Leiden, The Netherlands


USNMNational Museum of Natural History, Smithsonian Institution, Washington, D. C., USA


ZMANZoölogisch Museum Amsterdam, Leiden, The Netherlands


Whole male abdomens were removed from the body with fine forceps and treated with10% KOH solution at room temperature for 8–12 hours. Male terminalia were prised apart,rinsed and examined in 70% ethanol. Tegmina were photographed when totally dry in the air, while other parts of male terminalia were photographed in glycerol. All male terminalia components were permanentlystored within glycerol in genital vial which was pinned below specimen. Habitus images were captured using a Nikon D7000 digital camera with Tamron SP 90mm lens, or Canon 450D digital camera with CanonMacro 100 mm lens. Terminalia images were captured by a Nikon digital Sight DS–SM camera fitted to a Nikon SMZ–1500 stereoscopic dissecting microscope controlled by ACT–2U software, or by a Canon 450D digital camera fittedto a Nikon SMZ–1500 stereoscopic dissecting microscope. Series of partially focused photographswere taken and then combined using Helicon Focus software, and finallyprocessed with Adobe Photoshop software. Line drawings were made under Leica MZ125 stereoscopic dissecting microscope or created from color photographs using Adobe Illustrator software. Distribution maps, created in Adobe Illustrator software, are based on examined materials and published records.

Measurements were made under a stereomicroscope using an ocular micrometer. Body length is the linear distance from labrum to elytral apices. Body width is themaximum width across elytra. Abbreviations are shown in the text as follows: **AL**: antennal length; **AD**: distance between two antennae insertions. **EyD**: minimum distance between two eyes; **EyW**: maximum eye width in dorsal view (Fig. 71); **PL**: prothorax maximum length; **PW**: prothorax maximum width; **EL**: elytra maximum length; **EW:** elytra maximum width. Terminology mostly follows Ekis (1977). The male tegmen possesses three membranous semi-transparent regions, a pair of slit-like ones situated at both dorsal-lateral sides (Figs 59–60, 66–67) and the other, more or less cordiform, situated ventrally (Figs 60–61, 67–69); these membranous regions are more clear to see when tegmen is dry. The ventral membranous region is cordiform, surrounded by a pair of **outer margins** and a pair of **inner margins (**Figs 68, 69). Spicular forks are comprised of a spicular apodeme (*sensu* Opitz 2010) and a pair of spicular arms(Fig. 63; = lateral plates of the spicular fork *sensu* Opitz 2010). When describing male terminalia, the following abbreviations are introduced: **TML**: length of ventral membranous region of the tegmen; **TMW**: width of ventral membranous region of the tegmen (Figs 61, 68–69); **TMaL**: vertical length of apical lobe of the ventral membranous region of tegmen; **SApL**: length of spicular apodeme; **SFL**: length of spicular fork.

Original and later important taxonomic references are cited after taxon names. Full label data are provided for name-bearing type specimens: label data of each specimen are enclosed within a pair of double quotation marks, and individual labels are separated by a slash. All writings are cited in their original spelling, punctuation and language. Original italic or capital is ignored. Notes and elaborations relating to label data are enclosed in square brackets (including the writer, translation, etc). Red labels have been added to holotypes, paratypes, lectotypes and paralecto- types. Full label data or, in most cases, only locality data are provided for other specimens. When transcribing the label data, “**hw.**” is short for “handwritten”, and ellipsis are used if the original writing were illegible and unable to be transcribed. Authors of the handwriting on determination labels are identified with the clues given by Horn *et al*. (1990) and/or confirmed by present curators of correlative museums where the authors of those handwritings worked. Specimens marked with an asterisk are those whose male terminalia are figured in this paper.

## Taxonomy

### Callimerus latifrons

species-group

Brachycallimerus Chapin, 1924: 180, 190 (Type species: *Callimerus latifrons* Chapin, 1924; by original designation); –Kolibáč 1998: 176 (synonymized with *Callimerus* Gorham).
Crassocallimerus Pic, 1929: 16 (Type species: *Callimerus latesignatus* Gorham, 1892; by monotypy; subgenus of *Callimerus* Gorham); –Corporaal 1937: 60 (synonymized with *Brachycallimerus* Chapin).
flavofasciatus -group Kolibáč 1998: 182.

#### Diagnosis.

This species group is characteristic by its broad and compact body form. It differs from *dulcis* species-group (*sensu* Kolibáč 1998) and *prasinatus* species-group (*sensu* Kolibáč 1998) by pronotum wider than long (PL/PW < 1; Figs 4, 15, 17, 28, 30, 41, 44, 54); antenna short (AL/AD 1.0–1.2); antennomeres VII – XI or VIII – XI forming a more or less compact and oval club, width of antennomere VIII longer than or as long as its length (Figs 14, 27, 40, 53, 71); eyes large, posterior inner margins of eyes evidently convergent towards midline, EyD subequal to EyW (ratio 1.0–1.1) (Figs 4, 15, 17, 28, 30, 41, 44, 54); elytra without scales; integument with yellow and black coloration.

It differs from *coomani*-group (*sensu* Kolibáč 1998) by claws with a basal tooth (Fig. 57) and metatibiae with a subapical projection on the outer edge (Fig. 58).

In the integumental coloration (yellow and black), *Callimerus pallidus* Gorham, 1892 (Carin Hills, Chebà; Figs 79), *Callimerus gorhami* Corporaal, 1949 (Sumatra’s East Coast; Fig. 81), *Callimerus nigroapicalis* Pic, 1955 (Fujian; Fig. 84, 85), *Callimerus terminalis* Chapin, 1919 (Sandakan, North Borneo; Fig. 86, 87), and some species related to *Callimerus insolatus* Pascoe, 1860 might be similar to members of the *latifrons* species-group. The differences between *Callimerus pallidus*, *Callimerus gorhami* and this species group are provided in the text below. *Callimerus nigroapicalis* is different from this species group in pronotum longer than wide. *Callimerus terminalis* is different from this species group in claws without a basal tooth, subapical projection on outer edge of metatibia rudimental, pronotum longer than wide. Species related to *Callimerus insolatus* differ from this species group in claws without a basal tooth.

#### Description.

*Size*: length 7.5–11.7 mm, width 2.3–3.7 mm. *Integumental color*: yellow and black. *Vestiture*: body profusely vested with yellow pubescence; frons with dense white scales; thoracic pleuron sometimes with white scales. *Head*: including eyes wider than pronotum, vertex with sparse punctures. Labrum rectangular, apex straight or very slightly emarginated in the middle; mandibles stout; terminal segments of maxillary palpi digitiform, those of labial palpi elongate-triangular, both as long as their preceding segments. Eyes large, very slightly emarginated near antennal insertions, finely granulate; posterior inner margin of eyes evidently convergent towards midline, EyD subequal to EyW (ratio 1.0–1.1) (Figs 4, 15, 17, 28, 30, 41, 44, 54). Antenna short (AL/AD 1.0–1.2); antennomere I stout and bent, twice length as antennomere II; antennomere II globular; antennomere III longer than it is wide, slightly longer than antennomere II; antennomeres IV–X increasingly wider and shorter than their preceding segments, antennomeres VII or VIII to XI wider than or as long as their respective length, forming a more or less compact and oval club (Figs 14, 27, 40, 53, 71). Gula oblong, gular sutures parallel. *Prothorax*: wider than long (PL/PW 0.8–0.9), subapical impression of pronotum deep; pronotum constricted at base, evidently dilated before middle (Figs 4, 15, 17, 28, 30, 41, 44, 54); punctures on pronotum sparse and fine. *Elytra*: wider than head including eyes, EL/EW 1.7–2.3; sides parallel, sutural angle round, outer angle pointed (Fig. 70); punctures irregular and dense; without scales. *Legs*: tibiae without longitudinal ridge; metatibia with a subapical projection on outer edge (Fig. 58); tibial spur formula 0–1–1; tarsi formula 5–5–5, tarsomeres I–II evidently bilobed, III–IV more or less bilobed, V slender; tarsomere I of pro- and meso-tarsi as long as tarsomere II, meta-tarsomere I slightly longer than tarsomere II; tarsomere I–IV of all legs with evident pulvilli; claws with a basal tooth (Fig. 57). *Abdomen*: with six ventrites; male ventrite V–VI with posterior margin emarginated (Figs 13, 26, 39, 52, 65); female ventrite V with posterior margin straight, ventrite VI with posterior margin rounded. *Male terminalia*: tegmen tubular, sclerotized with three semi-transparent membranous regions: a pair of slit-like ones situated at dorsal-lateral sides (Figs 59–60, 66–67); the other one more or less cordiform, situated ventrally (Figs 60–61, 67–69). Outer margin of the ventral membranous region straight (Figs 9, 9a), slightly curved (Figs 22, 22a, 48, 48a, 61, 68, 69) or strongly curved (35, 35a); TML/TMW 0.9–1.3, TMaL/TML 0.2–0.6 (Figs 9, 9a, 22, 22a, 35, 35a, 48, 48a, 61, 68, 69). In some cases, an additional tiny membranous region presents at dorso-central side of tegmen (Fig. 66). Parameres strongly sclerotized, apices divergent (Figs 7–9, 59–61) or convergent (Figs 20–22, 33–35, 46–48). Phallic plate sclerotized in midline (Figs 10, 36, 49). Spicular fork: SApL/SFL about 0.3 (Figs 11, 24, 37, 50, 63).

#### Distribution.

Southeast Asia (Fig. 72).

#### Discussion.

This species group is probably advanced groups within *Callimerus* s. l., as Kolibáč (1998) suggested; but its sister group cannot be determined with certainty until the intra-taxonomy of the *dulcis* species-group (*sensu* Kolibáč 1998) has been resolved, which nearly contains two-thirds of species of this genus.

#### Key to species of *latifrons* species-group

**Table:** 

1	Elytron with only one black spot at apex (Fig. 54); metasternum and metepisternum both yellow*Callimerus cacuminis* sp. n.
-	Elytron with two black spots, anterior spot just before middle, posterior spot at apex or near apex (Figs 1, 15, 28, 41); metasternum and metepisternum both black	2
2	Head black; anterior black spot of elytron spanning from outer margin to suture or almost to suture (Figs 1, 2, 15, 17, 18)	3
-	Head yellow; anterior black spot of elytron clearly reaching neither outer margin or suture (Figs 28, 30, 31, 41, 42, 44)	4
3	Pronotum totally black; mesepisternum black; anterior black spot of elytron extended to suture thus forming a complete black band across elytra (Figs 1, 2); EL/EW about 2.2; apices of parameres divergent (Figs 7–9), outer margin of ventral membranous region of tegmen straight (Figs, 9, 9a)*Callimerus latifrons* Gorham
-	Pronotum with major area yellow, only with a small transverse black patch on anterior margin (Fig. 15), in few cases such patch absent (Fig. 17); mesepisternum yellow; anterior black spot of elytron not exactly extended to suture, thus forming a incomplete black band across elytra interrupted at suture (Figs 15, 17, 18); EL/EW about 1.7; apices of paramere convergent (Figs 20–22), outer margin of ventral membranous region of tegmen slightly curved (Figs 22, 22a)	*Callimerus latesignatus* Gorham
4	Mesepisternum yellow; posterior black spot of elytron located vertical-apically, rounded rectangle, length to width ratio about 1.5:1 (Fig. 28); outer margin of ventral membranous region of tegmen strongly curved, TML/TMW about 0.9 (Figs 35, 35a); tergite VIII with posterior margin rounded, slightly notched in middle (Fig. 38)	*Callimerus pectoralis* Schenkling
-	Mesepisternum black; posterior black spot of elytron located lateral-apically, bar-shaped, length to width ratio about 4:1 (Fig. 41); outer margin of ventral membranous region of tegmen slightly curved, TML/TMW about 1.3 (Figs 48, 48a); tergite VIII with posterior margin almost straight (Fig. 51)	*Callimerus rusticus* Gorham

### Callimerus latifrons

Gorham, 1876

http://species-id.net/wiki/Callimerus_latifrons

Figs 1–14 72

latifrons Gorham, 1876: 67 (*Callimerus*; type locality: “Philippines”); –Chapin 1924: 190 (*Brachycallimerus*); –Kolibáč 1998: 176 (*Callimerus*).
flavofasciatus Schenkling, 1902: 320 (*Callimerus*; type locality: “Siam”); –Schenkling 1916: 220 (Singapore); –Chapin 1924: 190 (synonymized with *latifrons* Gorham); –Corporaal 1924: 196 (synonym of *latifrons* Gorham); –Corporaal 1939: 193 (variety of *latifrons* Gorham); –Corporaal 1948: 287 (raised to species rank). **Syn. n.**

#### Type material examined.

**Figures 1–14.**
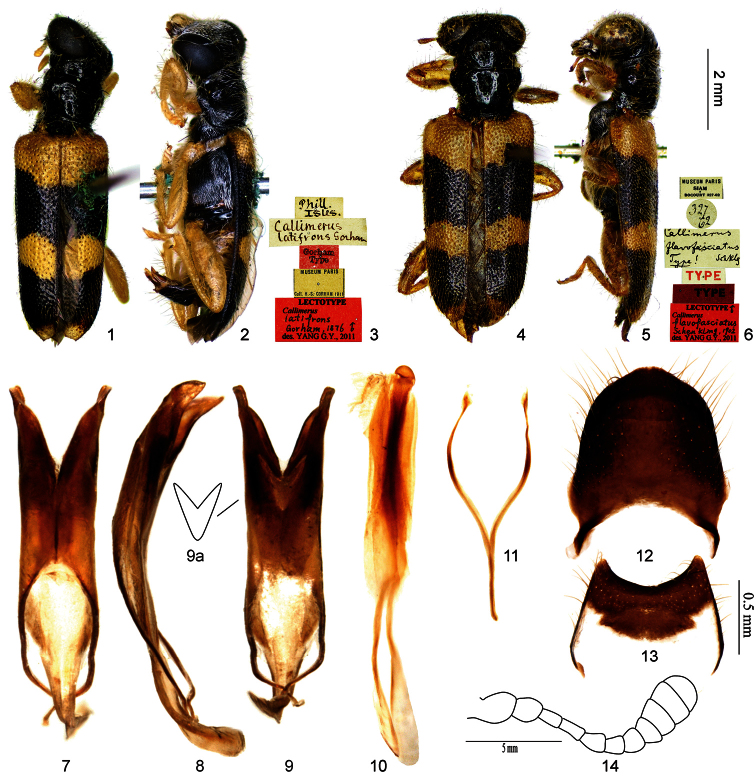
*Callimerus latifrons* Gorham, 1876. **1–3** lectotype of *Callimerus latifrons* Gorham, 1876 (**1** dorsal view **2** mirror image of right lateral view **3** labels) **4–6** lectotype of *Callimerus flavofasciatus* Schenkling, 1902 (**4** dorsal view **5** lateral view **6** labels) **7–13** male terminalia, specimen from Philippines **7–9** tegmen (**7** dorsal view **8** lateral view **9** ventral view **9a** outline of ventral membranous region) **10** phallus **11** spicular fork **12** tergite VIII **13** sternite VIII **14** antenna, specimen from Yunnan.

**Lectotype** of *Callimerus latifrons* Gorham designated here: “Phill. Isles / Callimerus latifrons Gorham [hw. by Gorham] / ♂ / Gorham Type / Museum Paris, Coll. H.S. Gorham 1911 / Lectotype: Callimerus latifrons Gorham, 1876 ♂, des. Yang G. Y., 2011” (MNHN, ♂; Figs 1–3); **Lectotype** of *Callimerus flavofasciatus* Schenkling designated here: “327–62 / Museum Paris; Siam; Bocourt 327-62 / Callimerus flavofasciatus Schklg. Type! [hw. by Schenkling] / Type / ♂ / Type / Lectotype ♂: Callimerus flavofasciatus Schenkling, 1902, des. Yang G. Y., 2011” (MNHN, ♂; Figs 4–6).

#### Note on Type material.

The name-bearing types of *latifrons* and *flavofasciatus* were not fixed in the original publications, so lectotypes of both species are designated here to express the taxonomic purpose of fixing the name to a single specimen and preventing further uncertainty regarding the taxon to which the names are applied. Only one specimen of each species was found in related museums.

#### Comment on synonymy.

Gorham (1876: 67) described *Callimerus latifrons* from Philippines as “Nigro-piceus, …, elytrorum fasciâ basali, maculâque pone medium reniformi pallide testaceis” (with “a basal fascia (widest in centre) and two kidney-shaped spots, almost touching suture, yellow”). Schenkling (1902: 320) published *Callimerus flavofasciatus* from Thailand, and stated that it differed from *Callimerus latifrons* by having an additional yellow spot at apex of elytron. Chapin (1924: 190) synonymized *Callimerus flavofasciatus* with *Callimerus latifrons* and argued, *Callimerus latifrons* having such a yellow spot at apex of elytron because all the Philippines specimens he examined having that (he also stated such a spot was mentioned in Gorham’s original description, which is not true though). Corporaal (1939: 193) found a specimen from Laos lacking a yellow spot at apex of elytron, which agrees well with Gorham’s description; so he regarded the specimen as a representative of the typical form of *Callimerus latifrons*, and treated *flavofasciatus* as a variety of *Callimerus latifrons* for having an additional yellow spot at elytral apex. Corporaal (1948: 287) re-treated *Callimerus flavofasciatus* as a distinct species for presence of such a spot. However, after we located it from MNHN, we found that the type of *Callimerus latifrons* actually having a yellow spot at apex of elytron, which was simply not mentioned in the original description. We compared the external morphology and male genital characters of type specimensof *Callimerus latifrons* and *Callimerus flavofasciatus* and found no significant differences; therefore, we synonymize *Callimerus flavofasciatus* with *Callimerus latifrons*. In addition, the specimen from Laos mentioned in Corporaal (1939: 193; 1948: 287) lacking the additional yellow spot is conspecific with *Callimerus latifrons*; it is only the color variation of this species, as posterior black bands of elytra reach to the extreme apex and thus the apical yellow spot missing.

#### Diagnosis.

*Callimerus latifrons* can be rapidly distinguished from other species of this species group by its entirely black pronotum (Figs 1, 2).

There are 3 other species of this species group with two black spots on each elytron: *Callimerus latesignatus*, *Callimerus pectoralis* and *Callimerus rusticus*. In addition to the difference in pronotal coloration, *Callimerus latifrons* can be differentiated from these three species by: (1) EL/EW about 2.2 (in other 3 species 1.7–1.8); (2) anterior black spot of elytron spanning from elytral outer margin to suture, thus forming a complete black band across elytra (Fig. 1); (3) apices of paramere divergent (Fig. 7–9).

#### Description.

*Size*:length 7.5–8.9 mm, width 2.3–2.8 mm. *Color*: Head black, clypeus, labrum, palpi and antennae yellow; pronotum black; elytron yellow with two black spots, anterior spot just before middle, posterior spot near apex or at apex, both spots spanning from elytral outer margin to suture, thus forming two complete black bands across elytra; legs yellow with metacoxae black; prosternum black; mesepisternum black, mesepimeron yellow, mesosternum black with anterior and posterior areas more or less yellowish; metepisternum, metasternum and katepisternum black; abdominal ventrites I–III of male yellow, IV–VI black; abdominal ventrites I–V of female yellow, VI black. *Vestiture*: meso- and meta- pleuron more or less with thin white scales. *Head*: AL/AD about 1.0; EyD/EyWabout 1.0. *Prothorax*: PL/PW about 0.9. *Elytra*: EL/EW about 2.2. *Male terminalia*: apices of paramere divergent (Figs 7–9); TML/TMW about 1.2, TMaL/TML about 0.4, outer margin of ventral membranous region straight (Figs 9, 9a); SApL/SFL about 0.3 (Fig. 11); tergite VIII with posterior margin rounded and slightly pointed (Fig. 12); sternite VIII with posterior margin roundly concave (Fig. 13).

**Variation**. The posterior black spot on elytron in most cases doesn’t reach to the extreme apex and thus a small region of elytron extreme apex is yellow. But in a few specimens the black spot reach to the extreme apex, so the yellow portion is missing. These two color forms could be found in the same locality (Yunnan, China for instance) and they are not correlated with sex.

**Other material examined. China:** **Yunnan:** Xishuangbanna Damenglong, 650 m, 1958.IV.9, WANG Shuyong, IOZ(E)1126312 (IZAS, 1♂); same data but 1958.V.5, PU Fuji, IOZ(E)1126311 (IZAS, 1♀); Xishuangbanna, Yunjinghong, 650 m, 1959.V.5, ZHANG Xuezhong, IOZ(E)1126310 (IZAS, 1♂); Xishuangbanna, Mengla, 620–650 m, 1959.V.13, PU Fuji, IOZ(E)1126319 (IZAS, 1♀); Xishuangbanna, Xiaomengyang, 850 m, 1958.VIII.18, ZHANG Yiran, IOZ(E)1126309 (IZAS, 1♀); same data but 1957.VI.10, WANG Shuyong, IOZ(E)1126306 (IZAS, 1♂); Jinping, Mengla, 420 m, 1956.IV.19, HUANG Keren et. al., IOZ(E)1126283 (IZAS, 1♀); Jinping, Mengla, 420 m, 1956.IV.27, HUANG Keren et. al., IOZ(E)1126284 (IZAS, 1♂); Jinping, Mengla, 370 m, 1956.IV.12, HUANG Keren et. al., IOZ(E)1126287 (IZAS, 1♂); **Vietnam:** Tonkin, Backan; Lemée, 1908; Musum Paris 1952, Coll. R. Oberthür (MNHN, 1♀); **Thailand:** nr. Chiangdao cave, 800 m, N. Thailand, 19-IV-1983, T. Shimomura leg. (MNHN, 1♀); **Philippines:** “L. Laglaize 1879; Dumalon Zamboanga; Mindanao / Callimerus latifrons Gorh., det. Corporaal 1923 / Musum Paris 1952, Coll. R. Oberthür / ♂” (MNHN, 1♂)^*^; **Malaysia**: “Doherty / Perak / Fry Coll. 1905. 100 / C. latifrons G. [hw. by Gorham] / Callimerus latifrons Gorham, Gorham det. [hw. by Gahan]” (NHML, 1♀); **Indonesia**: “J.B. Corporaal; Sumatra’s O. K. Medan, 24.12.1917; 20 M / Musum Paris 1952, Coll. R. Oberthür / ♂” (MNHN, 1♂); “J.B. Corporaal; Sumatra’s O. K. Medan, 24.12.1917; 20 M / Callimerus flavofasciatus Schenkling; Corporaal det. 1923 / Musum Paris 1952, Coll. R. Oberthür / ♂” (MNHN, 1♂); “J.B. Corporaal; Sumatra’s O. K. Medan, 24.12.1917; 20 M / Callimerus flavofasciatus Schl. / [...] / [...] / Corporaal det. 1923 / Musum Paris 1952, Coll. R. Oberthür / ♀” (MNHN, 1♀); “J.B. Corporaal; Sumatra’s O. K. Pagar Marbau, 15 / 12.’18; 24 M / J.B. Corporaal det. 1922: Callimerus flavofasciatus Schl. / Musum Paris 1952, Coll. R. Oberthür / ♂” (MNHN, 1♂); “J.B. Corporaal; Sumatra’s O. K. Pagar Marbau, 15 / 12.’18; 24 M / B. flavofasciatus Schl. / Musum Paris, Coll. M. Pic / ♂” (MNHN, 1♂); “J.B. Corporaal; Sumatra’s O. K. Medan, 1.11.[19]21; 20 M / J.B. Corporaal det. 1922: Callimerus flavofasciatus Schl. / Musum Paris 1952, Coll. R. Oberthür / ♂” (MNHN, 1♂); “J.B. Corporaal; Sumatra’s O. K. Medan, 6.1921; 20 M / 1938 J.B. Corporaal det: Brachycallimerus latifrons Gorh. var. flavofasciatus Schenkl. / Musum Paris 1939, Corporaal / ♂” (MNHN, 1♂); “J.B. Corporaal; Sumatra’s O. K. Medan, 22.1.[19]21; 20 M / 1938 J.B. Corporaal det: Brachycallimerus latifrons Gorh. var. flavofasciatus Schenkl. / Musum Paris, Coll. M. Pic / ♂” (MNHN, 1♂); “J.B. Corporaal; Sumatra’s O. K. Pagar Marbau, 15 / 12.’18; 24 M / Musum Paris 1952, Coll. R. Oberthür / ♀” (MNHN, 1♀); “J.B. Corporaal; Sumatra’s O. K. Pagar Marbau, 15 / 12.’18[1918]; 24 M / [...1] / Brachycallimerus flavofasciatus Schl. / Musum Paris, Coll. M. Pic / ♀” (MNHN, 1♀); “Sumatra, Si-Rambe, XII.90-III.91, E. Modigliani / 1941 Corporaal det. Brachycallimerus latifrons Gorh. var. flavofasciatus Schenkling” (MCSN, 1♀); “59192 / Doherty / Borneo, Pengaron / Fry Coll. 1905. 100 / C. latifrons G. [hw. by Gorham] / Callimerus latifrons Gorham, Borneo, Perak” (NHML, 1♀).

#### Distribution

(Fig. 72). China (Yunnan), Vietnam, Thailand, Philippines (Mindanao), Malaysia, Indonesia (Sumatra, Borneo).

### Callimerus latesignatus

Gorham, 1892

http://species-id.net/wiki/Callimerus_latesignatus

Figs 15–27 72

latesignatus Gorham, 1892: 728 (*Callimerus*; localities: “Carin Hills (Chebà)”, “Assam, Naga Hills”); –Chapin 1924: 190 (*Brachycallimerus*); –Kolibáč 1998: 176 (*Callimerus*).

#### Type material examined.

**Figures 15–27.**
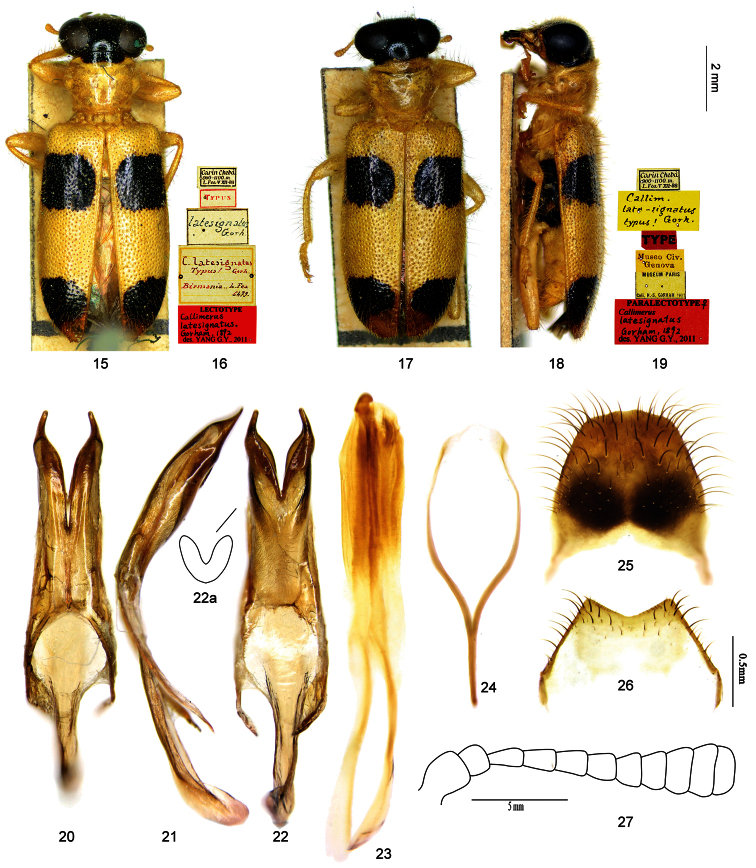
*Callimerus latesignatus* Gorham, 1892. **15–16** lectotype of *Callimerus latesignatus* Gorham, 1892 (**15** dorsal view **16** labels) **17–19** Paralectotype of *Callimerus latesignatus* Gorham, 1892 from MNHN (**17** dorsal view **18** lateral view **19** labels) **20–26** male terminalia, specimen from Yunnan **20–22** tegmen (**20** dorsal view **21** lateral view **22** ventral view **22a** outline of ventral membranous region) **23** phallus **24** spicular fork **25** tergite VIII **26** sternite VIII **27** antenna, specimen from Yunnan.

**Lectotype** of *Callimerus latesignatus* Gorham designated here:“Carin Chebà, 900–1000 m; L. Fea, V XII-88 / Typus / latesignatus Gorh. [hw. by Raffaello Gestro]/ Lectotype: Callimerus latesignatus Gorham, 1892, des. Yang G. Y., 2011” (MCSN, sex unknown; Figs 15–16); **Paralectotypes** of *Callimerus latesignatus* Gorham: “Carin Chebà, 900–1000 m; L. Fea, V XII-88 / Callim. late-signatus Gorh. typus! [hw. by Raffaello Gestro] / Type / Museo Civ. Genova / Museum Paris, Coll. H.S. Gorham, 1911 / Paralectotype ♀, Callimerus latesignatus Gorham, 1892, des. Yang G. Y., 2011” (MNHN, 1♀; Figs 17–19); “Doherty / Assam, Nagas / Fry Coll. 1905. 100. / C. latesignatus Gorh. [hw. by Gorham] / Callimerus latesignatus Gorham; Gorham det. [hw. by Gahan]” (NHML, 1 ex., sex unknown); “62022 / Doherty / Assam, Nagas / Fry Coll. 1905. 100. / Callimerus latesignatus Gorh., Assam, Type [“Type” with strikethrough; hw. by Frey]” (NHML, 1 ex., sex unknown).

#### Note on Type material.

The name-bearing type of *Callimerus latesignatus* was not fixed in the original publication so the lectotype is designated here to express the taxonomic purpose of fixing the name to a single specimen and preventing further uncertainty regarding the taxon to which the name is applied. The specimen deposited in MCSN is chosen as the lectotype because the type series were originally from that museum’s expedition.

#### Diagnosis.

*Callimerus latesignatus* is most similar to *Callimerus latifrons* and *Callimerus pectoralis*. It differs from *Callimerus latifrons* by: (1) pronotum with major area yellow, only with a small transverse black patch on anterior margin (Fig. 15), in few cases such patch absent (Fig. 17) (pronotum totally black in *Callimerus latifrons*); (2) mesepisternum yellow (black in *Callimerus latifrons*); (3) anterior black spot of elytron not exactly extended to suture, thus forming a incomplete black band across elytra interrupted at suture (Figs 15, 17, 18) (anterior black spot of elytron extended to suture in *Callimerus latifrons*, thus forming a complete black band across elytra; Figs 1, 2); (4) EL/EW about 1.7 (*Callimerus latifrons* with EL/EW about 2.2); (5) apices of paramere convergent (Figs 20–22) (*Callimerus latifrons* with apices of paramere divergent; Figs 7–9); (6) outer margin of ventral membranous region of tegmen slightly curved, TMaL/TML about 0.6 (Figs 22, 22a) (*Callimerus latifrons* with outer margin of ventral membranous region of tegmen straight, TMaL/TML about 0.4; Figs 9, 9a).

**Figures 28–40.**
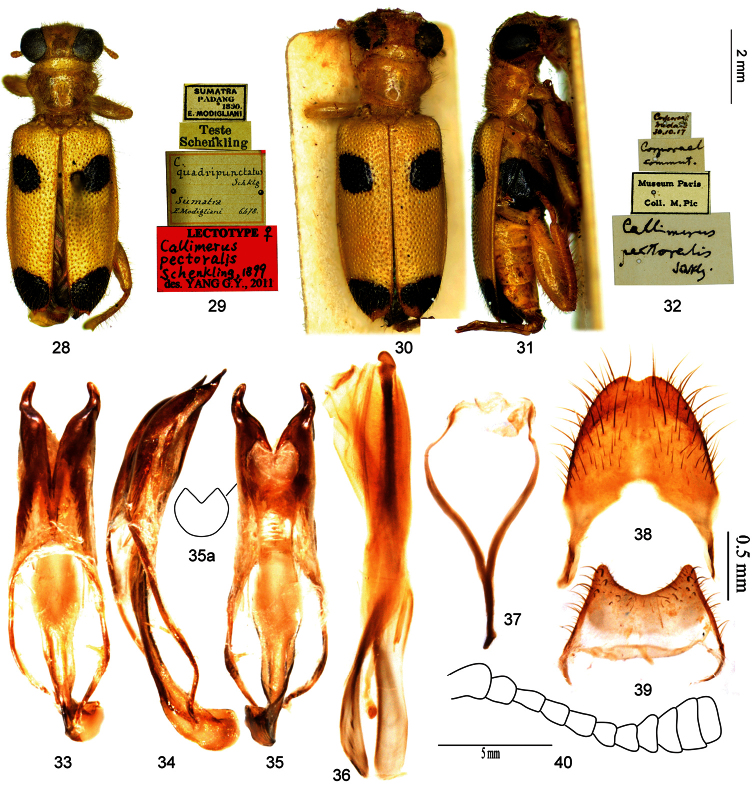
*Callimerus pectoralis* Schenkling, 1899. **28–29** lectotype of *Callimerus pectoralis* Schenkling, 1899 (**28** dorsal view **29** labels) **30–32** a specimen determined by Schenkling as *Callimerus pectoralis* (**30** dorsal view **31** lateral view **32** labels) **33–39** male terminalia, specimen from Sumatra **33–35** tegmen (**33** dorsal view **34** lateral view **35** ventral view **35a** outline of ventral membranous region) **36** phallus **37** spicular fork **38** tergite VIII **39** sternite VIII **40** antennae, specimen from Yunnan.

*Callimerus latesignatus* differs from *Callimerus pectoralis* by: (1) head black (*Callimerus pectoralis* with head yellow); (2) pronotum with major area yellow, only with a small transverse black patch on anterior margin (Fig. 15), in few cases such patch absent (Fig. 17)(*Callimerus pectoralis* with pronotum always totally yellow); (3) mesepisternum yellow (black in *Callimerus pectoralis*); (4) anterior spot of elytron spanning from elytral outer margin to nearly suture (Figs 15, 17, 18) (*Callimerus pectoralis* with anterior spot of elytron clearly neither reaching outer margin nor suture, such spot smaller; Figs 28, 30, 31); (5) outer margin of ventral membranous region of tegmen slightly curved, TMaL/TML about 0.6 (Figs 22, 22a) (*Callimerus pectoralis* with outer margin of ventral membranous region of tegmen strongly curved; TMaL/TML about 0.3; Figs 35, 35a); (6) tergite VIII with posterior margin almost straight (Fig. 25) (tergite VIII of *Callimerus pectoralis* with posterior margin notched in the middle; Fig. 38).

#### Description.

*Size*:length 8.2–10.5 mm, width 2.8–3.8 mm. *Color*: Head black, clypeus, labrum, palpi and antennae yellow; pronotum with major area yellow, only with a small transverse black patch on anterior margin (Fig. 15), in a few cases such patch absent (Fig. 17); elytron yellow with two black spots, anterior spot just before middle, posterior spot near apex, both spots spanning from elytral outer margin almost to suture, thus forming two incomplete bands across elytra interrupted at the suture; legs yellow with metacoxae mostly black; prosternum, mesepisternum, mesepimeron and mesosternum yellow; metepisternum, metasternum and katepisternum black; abdominal ventrites usually yellow, in a few cases terminal ventrite darker. *Head*: AL/AD about 1.0; EyD/EyWabout 1.0. *Prothorax*: PL/PW about 0.8. *Elytra*: EL/EW about 1.7. *Male terminalia*: apices of parameres convergent (Figs 20–22); TML/TMW about 0.9, TMaL/TML about 0.6, outer margin of ventral membranous region slightly curved (Figs 22, 22a); SApL/SFL about 0.3 (Fig. 24); tergite VIII with posterior margin almost straight (Fig. 25); sternite VIII with posterior margin shallowly triangularly concave (Fig. 26).

**Variation.** The pronotum of most specimens is yellow with a small transverse black patch on anterior margin, but two females examined don’t have such a patch and thus the pronotum is totally yellow (one of which is the paralectotype in MNHN).

#### Other material examined.

**China:** **Guangxi:** Daqingshan, Hengle, light trap, 1983.V.7, LIAO Subai (IZAS, 1♀); Pingxiang, YANG Jikun, 1963.V.10 (CAU, 3 ex.); same data but 1963.V.12 (CAU, 1 ex.); **Yunnan:** Mangshi, 900 m, 1955.V.16, Bustshik, IOZ(E)1126291 (IZAS, 1♂)^*^; Mangshi, 900 m, 1955.V.16, Kryzhanovskij, IOZ(E)1126292 (IZAS, 1♂); Mangshi, 900 m, 1955.V.16, Kryzhanovskij, IOZ(E)1126293 (IZAS, 1♀); Mangshi, 920 m, 1958.IX.1, LI Chuanlong, IOZ(E)1126325 (IZAS, 1♀); Gengma, 1955.V.2, HUANG Tianrong, IOZ(E)1126295 (IZAS, 1♀); eshan, 80.8, IOZ(E)1126959 (IZAS, 1♀); Baoshan, Diyidaoban, 1200 m, 1955.V.28, OU Bingrong, IOZ(E)1126305 (IZAS, 1♂); Baoshan to Yongping, 1955.V.28, B. Popov, IOZ(E)1126297 (IZAS, 1♀); Jingdong, Waidaba, 1250 m, 1956.V.26, YANG Xingchi, IOZ(E)1126289 (IZAS, 1♀); Jingdong, 1170 m, 1956.V.22, B. Popov, IOZ(E)1126300 (IZAS, 1♂); Jingdong, 1170 m, 1956.V.23, Kryzhanovskij, IOZ(E)1126301 (IZAS, 1♀); Jingdong, 1170 m, 1956.V.26, Kryzhanovskij, IOZ(E)1126302 (IZAS, 1 ex.); Jingdong,, 1200 m, 1955.IV.27, Kryzhanovskij, IOZ(E)1126303 (IZAS, 1 ex.); Jinping, Mengla, 420 m, 1956.IV.19, HUANG Keren et. al., IOZ(E)1126282 (IZAS, 1 ex.); Jinping, Mengla, 400 m, 1956.IV.27, HUANG Keren et. al., IOZ(E)1126285; IOZ(E)1126286 (IZAS, 2 ex.); Jinping, Mengla, 370 m, 1956.IV.22, HUANG Keren et. al., IOZ(E)1126288 (IZAS, 1 ex.); Cheli, Shihuiyao, 750 m, 1957.IV.27, D. Panfilov, IOZ(E)1126296 (IZAS, 1 ex.); Damenglong, 640 m, 1957.IV.28, WANG Shuyong, IOZ(E)1126304 (IZAS, 1 ex.); Xishuangbanna, Xiaomengyang, 850 m, 1957.VI.25, ZANG Lingchao, IOZ(E)1126307 (IZAS, 1 ex.); same data but 1957.III.28, ZANG Lingchao, IOZ(E)1126294 (IZAS, 1 ex.); same data but 1958.IX.7, ZHANG Yiran, IOZ(E)1126308 (IZAS, 1 ex.); same data but 1957.IV.2, WANG Shuyong, IOZ(E)1126290 (IZAS, 1 ex.); same data but 1000 m, 1957.V.6, ZANG Lingchao, IOZ(E)1126298 (IZAS, 1 ex.); Xishuangbanna, Meng’a, 1050–1080 m, 1958.V.16, IOZ(E)1126323 (IZAS, 1 ex.); same data but 1958.V.25, PU Fuji, IOZ(E)1126314 (IZAS, 1 ex.); same data but 1958.V.12, IOZ(E)1126324 (IZAS, 1 ex.); same data but 1050 m, 1958.V.20 IOZ(E)1126313 (IZAS, 1 ex.); Xishuangbanna, Mengzhe, 870 m, 1958.VII.7, PU Fuji, IOZ(E)1126315 (IZAS, 1 ex.); Xishuangbanna, Mengzhe, 870 m, 1958.IX.3, WANG Shuyong, IOZ(E)1126316 (IZAS, 1 ex.); Xishuangbanna, Mengla, 620–650 m, 1959.V.13, ZHANG Facai, IOZ(E)1126317 (IZAS, 1 ex.); same data but 1959.V.2, ZHANG Yiran, IOZ(E)1126318 (IZAS, 1 ex.); same data but 1959.VI.6, PU Fuji, IOZ(E)1126320 (IZAS, 1 ex.); same data but 1959.VI.6, ZHANG Yiran, IOZ(E)1126321 (IZAS, 1 ex.); same data but 1959.V.3, ZHANG Yiran, IOZ(E)1126322 (IZAS, 1 ex.); Hekou, Nanxi, Huayudong, 150 m, 2010.IV.27, ZHU Xiaoyu leg., under surface of leaves of Ficus (CCCC, 4 ex.); Honghe, Hekou, Nanxi, 150 m, 2009.V.21, LI Hu leg. (CAU, 1♀); **Vietnam:** “Museum Paris; Tonkin sept., Montagnes du Haut Song-Chai, Rabier 1895 / Museum Paris; Mes du Ht Song-Chai; Rabier 258-95 / Callimerus latesignatus Gorh., Schenkling vid. 1901” (MNHN, 1♂); “Museum Paris; Tonkin; Langue 1887 / Callimerus latesignatus Gorh., Schenkling vid 1901 / Compare au British Museum; P. Lesne 1907 / Callimerus latesignatus Gorh., P. Lesne vid.” (MNHN, 1♂); Tonkin occ., Env. de Hoa-Binh, R.P A. de Cooman 1919 (MNHN, 4 ex.); Tonkin, P. Lemée, 1903–1906 (MNHN, 3 ex.); **Laos**: Louang-Prabang, A Theng; A. Pavie 1888 (MNHN, 1 ex.); Laos (MNHN, 1 ex.); “Laos-NE, Xieng Khouang prov., 19°38'N, 103°20'E-19°37'N, 103°21'E, 30km NE Phonsavan: Ban Na Lam→Phou Sane Mt., 1300–1700 m, 10.-30.v.2009, M. Geiser leg. / NHMB Basel, NMPC Prague, Laos 2009 Expedition: M. Brancucci, M. Geiser, Z. Kraus, D. Hauck, V. Kuban” (NHMB, 1 ex.); “Laos-NE, Xieng Khouang prov.,~19°37–8'N 103°20'E, Phonsavan (30 km NE): Phou Sane Mt., ~1400–1500 m, 10.-30.v.2009, Z. Kraus leg. / NHMB Basel, NMPC Prague, Laos 2009 Expedition: M. Brancucci, M. Geiser, Z. Kraus, D. Hauck, V. Kuban” (NHMB, 1 ex.); **Thailand**: Siam, Lot 319, 3300 feet, 21 Jun 1936 (MNHN, 1 ex.); “Doi Suthep, 1100 m, Chiang Mai, N. Thailand, 15-IV-1983, T. Shimomura leg.” (MNHN, 1 ex.); “Mt. Doi Pui, 1400–1500 m, Chiang Mai, N. Thailand, 28-IV-1983, T. Shimomura leg.” (MNHN, 1 ex.); **India**: “Village 9th mile, nt. Rani Pul, 24.4 / Sikkim 77, Bhakta B.” (NHMB, 2 ex.); Khasia Hills, VI. 96 (MNHN, 1 ex.); “Mali 900 m, 28.4.1981 / Sikkim, Bhakta B.” (NHMB, 1 ex.); “Tista, 18.IV.1987 / Indien, Darjeeling D., Bhakta B.” (NHMB, 1 ex.); “Pudung, 24–25.V.87 / Indien, Darjeeling D., Bhakta B.” (NHMB, 1 ex.); “Pudung, 18.IV.1990 / Indien, Darjeeling D., Ch. J. Rai” (NHMB, 1 ex.); “Alghera, 2900 m, 25.IV.1982 / Darjeeling D., Ch. J. Rai” (NHMB, 1 ex.); “Umg. Kalimpong, Darjeeling Distr., 4.4.77” (NHMB, 1 ex.); “Kalimpong, Umg, Bhakta Bahadur, 10.5.77” (NHMB, 3 ex.); “Kalimpong 850 m, Nashay, 16.IV.1984 / Indien, Darjeeling D., Ch. J. Rai” (NHMB, 1 ex.); “Pudung, 24–25.V.87 / Indien, Darjeeling D., Bhakta B.” (NHMB, 1 ex.); **Bhutan:** British Bootang, L. Durel, 1899 (MNHN, 2 ex.); British Bootang, Maria Basti, 1900 (MNHN, 1 ex.); British Bootang, Maria Basti, 1899 (MNHN, 1 ex.); British Bootang, Padong, L. Durel 1913 (MNHN, 1 ex.); Pedong, A. Desgodins (MNHN, 2 ex.).

#### Distribution

(Fig. 72). China (Guangxi, Yunnan), Vietnam, Laos, Thailand, Myanmar, India, Bhutan.

### Callimerus pectoralis

Schenkling, 1899b

http://species-id.net/wiki/Callimerus_pectoralis

Figs 28–40 72

pectoralis Schenkling, 1899b: 335 (*Callimerus*; type locality: “Sumatra (Padang)”).

#### Type material examined.

**Lectotype** of *Callimerus pectoralis* Schenkling designated here: “Sumatra / Padang, 1890, E. Modigliani / Teste Schenkling / C. quadripunctatus Schklg, Sumatra, E. Modigliani, 6478 [hw. by Raffaello Gestro]/ Lectotype ♀: Callimerus pectoralis Schenkling, 1899, des. Yang G. Y, 2011” (MCSN, ♀; Figs 28–29).

#### Note on Type material.

The lectotype is one of the specimens sent to S. Schenkling for study from MCSN, with a label “quadripunctatus Schklg” handwritten by R. Gestro, the former curator of MCSN. The name “quadripunctatus Schklg” has never been published, however, the locality and morphological characters of this specimen perfectly accord with the original publication of *Callimerus pectoralis*. On the other hand, a specimen found in Coll. M. Pic from MNHN (locality Medan, Sumatra) determined as *Callimerus pectoralis* by Schenkling himself is conspecific with the specimen labeled “quadripunctatus Schklg” (Figs 30–32). In this case, it is assumed that “*quadripunctatus*” was the first name that came to Schenkling’s mind and written down in the identification list that sent to MCSN, but later Schenkling changed his mind and published the species with another name “*pectoralis*”. Regardless of the details, the specimen found in MCSN undoubtedly belongs to the type series and we therefore designated it as the lectotype of *Callimerus pectoralis* Schenkling here for the taxonomic purpose of fixing the name to a single specimen and preventing further uncertainty regarding the taxon to which the name is applied.

#### Diagnosis.

**Figures 41–53.**
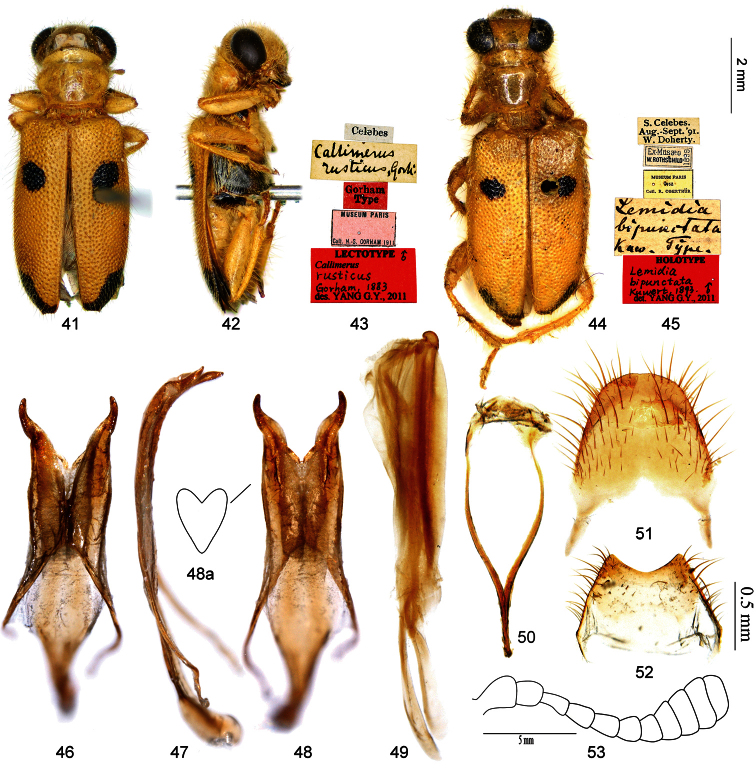
*Callimerus rusticus* Gorham, 1883. **41–43** Lectotype of *Callimerus rusticus* Gorham, 1883 (**41** dorsal view **42** lateral view **43** labels) **44–45** Holotype of *Lemidia bipunctatus* Kuwert, 1893 (**44** dorsal view **45** labels) **46–52** male terminalia, specimen from Celebes **46–48** tegmen (**46** dorsal view **47** lateral view **48** ventral view **48a** outline of ventral membranous region) **49** phallus **50** spicular fork **51** tergite VIII **52** sternite VIII **53** antennae, specimen from Celebes.

This species is most similar to *Callimerus rusticus* and *Callimerus latesignatus*. However, it differs from *Callimerus rusticus* by: (1) mesepisternum yellow (black in *Callimerus rusticus*); (2) posterior spot on elytron located vertical-apically, rounded rectangle, length to width ratio about 1.5:1 (Fig. 28)(*Callimerus rusticus* with posterior spot on elytron located lateral-apically, bar-shaped, length to width ratio about 4:1; Fig. 41); (3) anterior spot on elytron of *Callimerus pectoralis* larger than that of *Callimerus rusticus*, in *Callimerus pectoralis* short diameter of anterior spot on elytron in most cases larger than distance between that spot and elytral suture (Fig. 28, 30) (*Callimerus rusticus* short diameter of anterior spot on elytron in most cases not larger than distance between that spot and elytral suture; Fig. 41, 44); (4) outer margin of ventral membranous region of tegmen strongly curved, TML/TMW about 0.9, TMaL/TML about 0.3 (Figs 35, 35a) (*Callimerus rusticus* with outer margin of ventral membranous region of tegmen slightly curved, TML/TMW about 1.3, TMaL/TML about 0.2; Figs 48, 48a); (5) tergite VIII with posterior margin rounded, slightly notched in middle (Fig. 38) (*Callimerus rusticus* with tergite VIII with posterior margin almost straight; Fig. 51).

The difference between this species and *Callimerus latesignatus* is provided in the diagnosis section under *Callimerus latesignatus*.

#### Description.

*Size*:length 7.5–8.8 mm, width 2.8–3.3 mm. *Color*: Head yellow, mandibles black; pronotum yellow. Elytron yellow with two spots, anterior spot at basal one fourth, posterior spot at vertical-apex; anterior spot rounded rectangle, neither reaching outer margin nor suture, short diameter of anterior spot on elytron in most cases greater than distance between that spot and elytral suture (Fig. 28, 30); posterior spot rounded rectangle, length to width ratio about 1.5:1 (Fig. 28, 30). Legs yellow with metacoxae mostly black. Prosternum, mesepisternum, mesepimeron and mesosternum yellow; metepisternum, metasternum and katepisternum black; abdominal ventrites yellow. *Head*: AL/AD about 1.0; EyD/EyW about 1.0. *Prothorax*: PL/PW about 0.8. *Elytra*: EL/EW about 1.7. *Male terminalia*: apices of parameres convergent (Figs 33–35); TML/TMW about 0.9; TMaL/TML about 0.3, outer margin of ventral membranous region strongly curved (Figs 35, 35a); SApL/SFL about 0.3 (Fig. 37); tergite VIII with posterior margin rounded, slightly notched in middle (Fig. 38); sternite VIII with posterior margin roundly concave (Fig. 39).

#### Other material examined.

**China**: **Yunnan:** Xishuangbanna, Damenglong, 650 m, 1958.IX.17, PU Fuji leg., IOZ (E)1126329 (IZAS, 1♂); **Malaysia:** Perak, Doherty (MNHN, 1 ex.; NHML, 5 ex.); Malacca, Perak, W. Doherty (MNHN, 1 ex.); Perak, Malacca (Doherty) (MNHN, 1 ex.); “Penang / Bowring 63.47* / Callimerus pectoralis Scklg, S. Schenkling det.” (NHML, 2); “Penang / Ex. A. R. Wallace, Private collection, Purchased 1860–70, Ox. Uni. Mus. of Nat. Hist. (OUMNH)” (OUM, 4 ex.); Kuching, Sarawak, G.E. Bryant, 28.XI.13 (NHML, 1 ex.); **Indonesia:** J.B. Corporaal; Sumatra’s O. K., Medan, 1.11.20, 20 M (MNHN, 1♂)^*^; same data but 20.2.18 (MNHN, 1 ex.); same data but 8.2.21 (MNHN, 1 ex.); same data but 9.8.1921 (MNHN, 3 ex.); same data but 11.8.1921 (MNHN, 1); same data but 18.8.1921 (MNHN, 1 ex.); same data but 10.9.1921 (MNHN, 1 ex.); “Corporaal, Medan, 30.10.17 / Corporaal comment / Museum Paris, Coll. M. Pic / Callimerus pectoralis Schklg [hw. by Schenkling]” (MNHN, 1♂); Sumatra, Padang Sidempoean, XII.1902-I.1903 (MNHN, 2 ex.); “Dolok Merangir, 18.VII-9.X.1980 / Sumatra, Dr. E. Diehl” (NHMB, 2 ex.); “42618 / Borneo, Labuan / Fry Coll. 1905. 100 / C. rusticus G. [hw. by Gorham] / Callimerus rusticus Gorham, det. Borneo, Perak [hw. by Fry]” (NHML, 1 ex.); Sud Borneo, Goenong Pandjang, Tanggarang, M. E.Walsh, VI.1937 (MNHN, 1 ex.).

#### Distribution

(Fig. 72). China (Yunnan), Malaysia, Indonesia (Sumatra, Borneo).

### Callimerus rusticus

Gorham, 1883

http://species-id.net/wiki/Callimerus_rusticus

Figs 41–53 72

rusticus Gorham, 1883: 252 (*Callimerus*; localities: “Celebes”, “Sangir”).
bipunctatus Kuwert, 1893: 485 (*Lemidia*; type locality: “Celebes”); –Schenkling 1898: 169 (synonymized with *rusticus* Gorham).

#### Type material examined.

**Lectotype** of*Callimerus rusticus* Gorham designated here: “Celebes / Callimerus rusticus, Gorh. / Gorham Type / Museum Paris, Coll. H. S. Gorham, 1911/ Lectotype ♂: Callimerus rusticus Gorham, 1883, des. Yang G. Y., 2011” (MNHN, ♂; Figs 41–43); **Paralectotype** of*Callimerus rusticus*: “Rosenb., Sangir / Callimerus rusticus Gorham / Type ♀ / Type / rusticus Gorh. n. sp.” (RMNH, 1♀); **Holotype** of *Lemidia bipunctatus* Kuwert: “S. Celebes, Aug.-Sept. ‘91, W. Doherty / Ex-Musaeo, W. Rothschild, 1899 / Museum Paris, 1952, Coll. R. Oberthür / Lemidia bipunctata Kuw. Type / Holotype: Lemidia bipunctata Kuwert, 1893, ♂, det. Yang G. Y., 2011” (MNHN, ♂; Figs 44–45).

#### Note on Type material.

The original description of *Callimerus rusticus* Gorham mentioned two specimens, but the name-bearing type was not fixed. We found both syntypes in MNHN and RMNH respectively, and designate the male from Coll. Gorham in MNHN as lectotype here to express the taxonomic purpose of fixing the name to a single specimen and preventing further uncertainty regarding the taxon to which the name is applied.

The original publication of *Lemidia bipunctatus* Kuwert noted that only one specimen was examined, so the holotype was originally fixed by monotypy.

#### Diagnosis.

This species can be rapidly distinguished from other species of the *latifrons* species-group by posterior spot on elytron being located lateral-apically, bar-shaped, with a length to width ratio of about 4:1 (Fig. 41).

This species is most similar to *Callimerus pectoralis*; the difference between them is provided in the diagnosis section under *Callimerus pectoralis*.

#### Description.

*Size*:length 7.5–9.5 mm, width 2.4–3.0 mm. *Color*: Head yellow, mandibles black; pronotum yellow. Elytron yellow with two spots, anterior spot at basal fourth, posterior spot at lateral-apex; anterior spot round and small, clearly neither reaching outer margin nor suture, short diameter of anterior spot on elytron in most cases not greater than distance between that spot and elytral suture (Fig. 41, 44); posterior spot bar-shaped, length to width ratio about 4:1 (Fig. 41). Legs yellow with metacoxae mostly black. Prosternum yellow; mesepisternum black, mesepimeron and mesosternum yellow; metepisternum, metasternum and katepisternum black; abdominal ventrites yellow. *Head*: AL/AD about 1.0; EyD/EyW about 1.0. *Prothorax*: PL/PW about 0.8. *Elytra*: EL/EW about 1.8. *Male terminalia*: apices of parameres convergent (Figs 46–48); TML/TMW about 1.3; TMaL/TML about 0.22, outer margin of ventral membranous region slightly curved (Figs 48, 48a); SApL/SFL about 0.3 (Fig. 50); tergite VIII with posterior margin almost straight, sternite VIII with posterior margin shallowly concave (Figs 51, 52).

#### Other material examined.

Ost-Celebes, Tombugu, H. Kühn 1885 (MNHN, 1♂)^*^; Ost-Celebes, Tombugu, H. Kühn 1885 (MNHN, 1 ex.); W. Celebes, G. Rangkoenau, J.P. Ch. Kalis, 900’ 1937 (MNHN, 1 ex.); Celebes, Menado (MNHN, 1♀); “Celebes, G. Heinrich, B. M. 1933-117 / Celebes, Latimodjonggeb. Uru, 800 m,Aug / Sept.1930, G. Heinrich / Brachycallimerus pectoralis (Schenkling), det. G. Ekis, 1985” (NHML, 1 ex.); “Drs. Sarasin, S. Celebes, Makassar / Callimerus rusticus Gorh., Determ K. M. Heller” (NHMB, 8 ex.); “Men [Sulawesi, Manado] / Ex. A. R. Wallace, Private collection, Purchased 1860–70, Ox. Uni. Mus. of Nat. Hist. (OUMNH)” (OUM, 1 ex.).

#### Distribution

(Fig. 72). Indonesia (Sulawesi).

### Callimerus cacuminis

G.Y. Yang & X.K. Yang sp. n.

urn:lsid:zoobank.org:act:4F0856EE-E6E2-473C-86D7-05B84D1143F4

http://species-id.net/wiki/Callimerus_cacuminis

Figs 54 72

#### Holotype.

**Figures 54–71.**
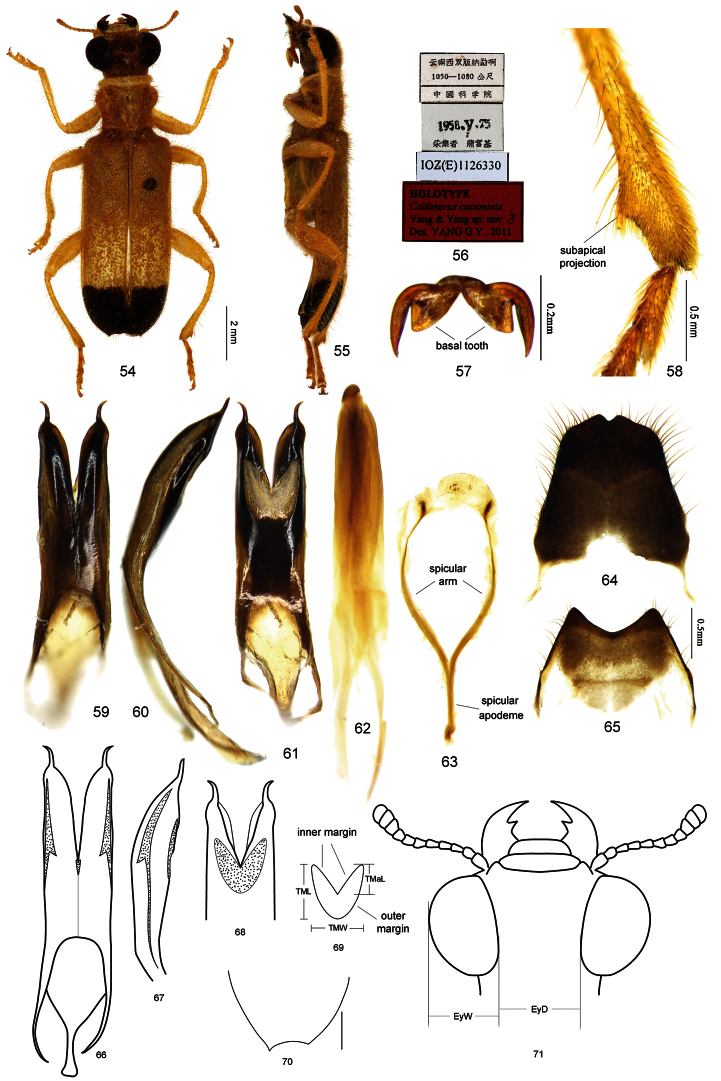
Holotypeof *Callimerus cacuminis* YANG & YANG. **54–55** habitus (**54** dorsal view **55** ventral view) **56** labels **57** claw **58** subapical metatibia **59**–**61** tegmen (**59** dorsal view **60** lateral view **61** ventral view) **62** phallus **63** spicular fork **64** tergite VIII **65** sternite VIII **66–69** tegmen (**66** dorsal view **67** lateral view **68** ventral view **69** outline of ventral membranous region) **70** elytron apex **71** head.

**China:** “云南西双版纳勐啊, 1050–1080公尺；中国科学院 [Yunnan,Xishuang- banna, Meng’a, 1050–1080 m] / 1958.V.25, 采集者: 蒲富基 [PU Fuji leg.] / IOZ(E)1126330 / Holotype: Callimerus cacuminis Yang & Yang sp. nov. ♂, Des. Yang G. Y., 2011” (IZAS, ♂; Figs 54–56)^*^; **Paratypes** (6 ex). **China**: same data as holotype, but 1958.VIII.17, IOZ(E)1126331 (IZAS, 1♀); same data but 1958.V.23, ZHANG Yiran leg., IOZ(E)1126332 (IZAS, 1♂); Yunnan, Xishuangbanna, Mengla, 620–650 m, 1959.V.30, ZHANG Yiran leg., IOZ(E)1126996 (IZAS, 1♂); Yunnan, Menglongbanna, Mengsong, 1600 m, 1958.VIII.8, WANG Shuyong leg., IOZ(E)1126333 (IZAS, 1♀); **Laos**: “Laos-NE, Xieng Khouang prov., 19°38'N, 103°20'E-19°37'N, 103°21'E, 30km NE Phonsavan: Ban Na Lam→Phou Sane Mt., 1300–1500 m, 10. –30.v.2009, M. Brancucci leg./NHMB Basel, NMPC Prague, Laos 2009 Expedition: M. Brancucci, M. Geiser, Z. Kraus, D. Hauck, V. Kuban” (NHMB, 1♀); “Laos-NE, Xieng Khouang prov.,~19°37–8'N 103°20'E, Phonsavan (30 km NE): Phou Sane Mt., ~1400–1500 m, 10.-30.v.2009, Z. Kraus leg./NHMB Basel, NMPC Prague, Laos 2009 Expedition: M. Brancucci, M. Geiser, Z. Kraus, D. Hauck, V. Kuban” (NHMB, 1♀).

#### Diagnosis.

The new species can be rapidly distinguished from other species of the *latifrons* species-group by: elytron with only one black spot at apex, lacking anterior spot; metasternum and metepisternum yellow; apices of parameres sharply attenuate, then divergent.

The coloration of this new species is similar to that of *Callimerus pallidus* Gorham and *Callimerus gorhami* Corporaal, which are excluded from the *latifrons* species-group in the present paper. The new species, however, differs from *Callimerus pallidus* by EL/EW 2.27–2.34, PL/PW about 0.9, EyD/EyW about 1.1 (Fig. 54) (EL/EW about 3.1, PL/PW about 1.2, EyD/EyW about 1.9 in *Callimerus pallidus*; Fig. 79).

The new species differs from *Callimerus gorhami* by: PL/PW about 0.9, EyD/EyWabout 1.1, elytra parallel-sided, without scales, apical black spot on elytron with anterior border straight (Fig. 54) (*Callimerus gorhami* with PL/PW about 1.1, EyD/EyW about 2.4, elytra slightly rounded at posterior sides, elytra apex with white scales, apical black spot with anterior border sinuate (Figs 81, 82)).

#### Description.

*Size*:length 9.9–11.7 mm, width 2.9–3.7 mm. *Color*: Head yellow, mandibles black; pronotum yellow; elytron yellow with a black spot at apex; legs yellow; under surface totally yellow except for last two ventrites of male abdomen and part of last ventrite of female abdomen darker. *Head*: AL/AD about 1.2 (Fig. 71); EyD/EyW about 1.1 (Fig. 71). *Prothorax*: PL/PW about 0.9. *Elytra*: EL/EW about 2.27–2.34. *Male terminalia*: apices of parameres sharply attenuate, slender and divergent (Figs 59–61, 66–68); TML/TMW about 1.1, TMaL/TML about 0.54, outer margin of ventral membranous region of tegmen curved (Figs 61, 68, 69); tegmen with an additional tiny membranous region at dorsal-central side (Fig. 66); SApL/SFL about 0.31 (Fig. 63); tergite VIII with posterior margin pointed, notched in middle (Fig. 64), sternite VIII with posterior margin deeply concave (Fig 65).

#### Variation.

In the holotype and a paratype (No. IOZ(E)1126332), the black spot on elytron reach to the extreme apex; in the other paratypes, the black spot does not reach to the extreme apex so that a tiny region in extreme apex is yellow.

#### Distribution

**Figures 72.**
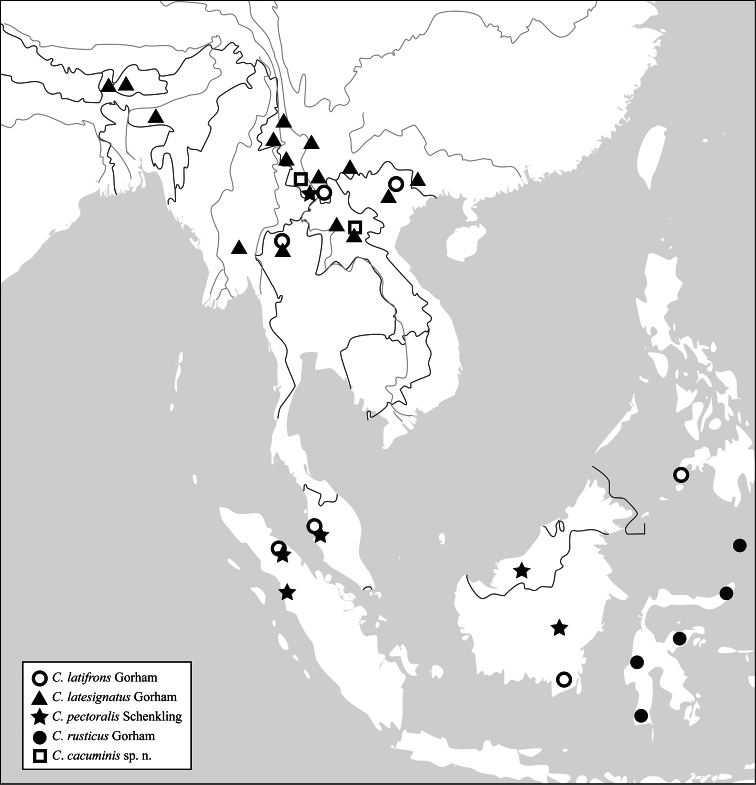
Distribution map of *Callimerus*, *latifrons* species-group.

(Fig. 72). China (Yunnan), Laos.

#### Etymology.

The Latin adjective “*cacuminis*” means of a peak, top or tip, and emphasizes the singular black spot on elytra apex.

### Species excluded from the *latifrons* species-group:

#### Corynommadius trifasciatus

(Schenkling, 1899a) comb. n.

http://species-id.net/wiki/Corynommadius_trifasciatus

Figs 73–75

trifasciatus Schenkling, 1899a: 136 (*Callimerus*; type locality: “Neu Guinea (Fly River)”); –Chapin 1924: 190 (“*Brachycallimerus*?”); –Corporaal 1937: 60 (*Brachycallimerus*).

##### Type material examined.

**Figures 73–83.**
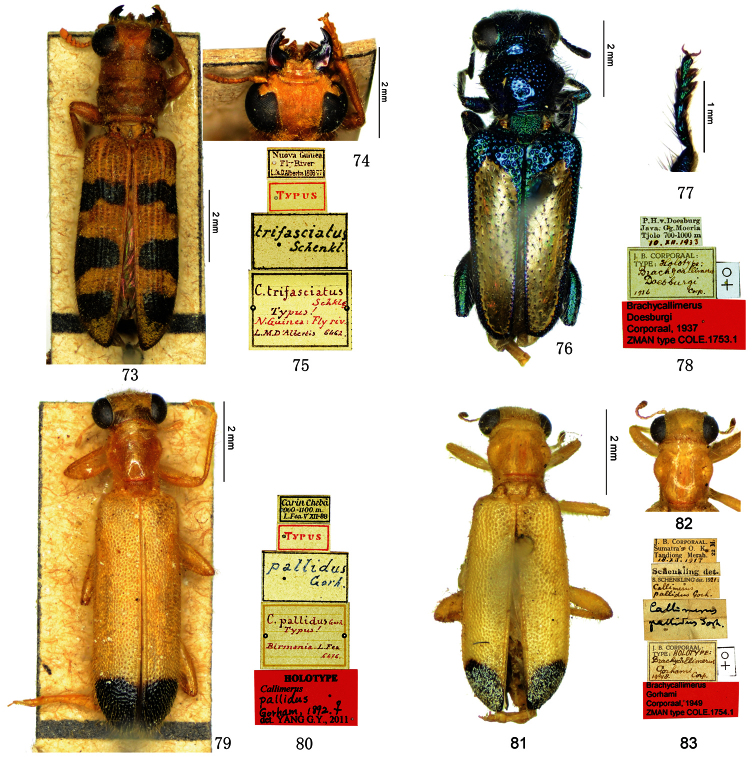
**73–75** Lectotype of*Callimerus trifasciatus* Schenkling, 1899 (**73** habitus **74** head **75** labels) **76–78** *Brachycallimerus doesburgi* Corporaal, 1937 (**76** habitus of holotype **77** hind leg of paratype **78** labels of holotype) **79–80** Holotype of *Callimerus pallidus* Gorham, 1892 (**79** habitus **80** label) **81–83 **Holotype of *Callimerus gorhami* Corporaal, 1949 (**81** habitus **82** head **83** labels).

**Lectotype of** *Callimerus trifasciatus* Schenkling designated here: “Nuova Guinea, Fly River, L. M. D. Albertis, 1876–77 / Typus / trifasciatus Schenkl. / C. trifasciatus Schklg, Typus! N. Guinea: Fly riv., L.M.D’Albertis, 6462 [hw. by Raffaello Gestro]” (MCSN, ♀; Figs 73–75).

##### Note on Type material.

The name-bearing type of *trifasciatus* was not fixed in the original publication. We designate the only specimen found in MCSN as lectotype to express the taxonomic purpose of fixing the name to a single specimen and preventing further uncertainty regarding the taxon to which the name is applied [The red lectotype label will be sent to MCSN after this paper published].

##### Taxonomic position.

This species is excluded from the genus *Callimerus* for its following characters: labrum deeply incised; eyes deeply emarginated near the antennal insertions; elytral punctures in rows; elytra clothed with unspecialized descent setae (not scales); the first tarsomeres very small and invisible from dorsal view; metatibia with 2 spurs in apex; metatarsi having pulvillus on tarsomere IV (in *Callimerus* labrum with apex straight or very slightly emarginated in the middle; eyes very slightly emarginated at antennal insertions; elytral punctures not in rows; elytra with scales or glabrous; the first tarsomeres of normal size, not shorter than the second tarsomeres; metatibia with 1 spur in apex; metatarsi having pulvilli on tarsomere I–IV). Furthermore, we consider it should be assigned in the genus *Corynommadius* Schenkling, 1899a. Its closest relative is *Corynommadius speciosus* Schenkling, 1899a, the type species of *Corynommadius* Schenkling; the difference of these two species only shows in variation of anterior black spot on elytron (Fig. 73; Gerstmeier 2002: 425, 432, fig. 2).

#### Callimerus doesburgi

(Corporaal, 1937)

http://species-id.net/wiki/Callimerus_doesburgi

Figs 76–78

doesburgi Corporaal, 1937: 60 (*Brachycallimerus*; type locality: “Java”); –Kolibáč 1998: 182 (*Callimerus coomani*-group).

##### Type material examined.

**Holotype** of *Brachycallimerus doesburgi* Corporaal: “P. H. v. Doesburg, Java, Gg. Moeria, Tjolo, 700–1000, 10.XII.1933 / J. B. Corporaal: Holotype: Brachycallimerus doesburgi Corp., 1936 [hw. by Corporaal] / Brachycallimerus doesburgi Corporaal, 1937, ZMAN type COLE. 1753. 1” (ZMAN, ♀; Figs 76–78); **Paratype** of *Callimerus doesburgi*: “Java merid, 1500, 1891, H. Fruhstorfer / J. B. Corporaal, Allotype: Brachycallimerus doesburgi Corp., 1936 [hw. by Corporaal] / Museum Paris, 1952, Coll. R. Oberthür / Type / Paratype: Brachycallimerus doesburgi Corporaal, 1937; det. Yang G. Y., 2011” (MNHN, 1♀).

##### Note on Type material.

The sex of the paratype was mistaken in the original publication.

##### Taxonomic position.

This species was included in Kolibáč’s (1998) “*coomani*-group” for its claws without a basal tooth; moreover, its metatibia without subapical projection on outer edge and body with metallic luster, are evidence in support of Kolibáč’s assignment.

#### Callimerus pallidus

Gorham, 1892 [Callimerus incertae sedis]

http://species-id.net/wiki/Callimerus_pallidus

Figs 79–80

pallidus Gorham, 1892: 727 (*Callimerus*; type locality: “Carin Hills (Chebà)”); –Corporaal 1937: 60 (*Brachycallimerus*).

##### Type material examined.

**Holotype** of *Callimerus pallidus* Gorham: “Carin Chebà, 900–1000 m, L. Fea V XII-88 / Typus / pallidus Gorh. [hw. by Gorham] / C. pallidus Gorh. Typus! Birmania, L. Fea, 6476 [hw. by Raffaello Gestro] / Holotype: Callimerus pallidus Gorham, 1892, ♀, det. Yang G. Y., 2011” (MCSN, ♀; Figs 79–80).

##### Note on Type material.

The original publication of *Callimerus pallidus* noted that only one specimen was examined, so the holotype was fixed in the original publication by monotypy.

##### Taxonomic position.

Although this species has a basal tooth on claw, and we didn’t examine its character of metatibia subapical projection (the only examined specimen is glued on board with metatibia not viewable), we are still confident to exclude it from the *latifrons* species-group for the following character states: PL/PW >1 (ratio 1.2), EyD evidently larger than EyW (ratio 1.9). Its nearest relative within the large genus *Callimerus* is still unclear.

#### Callimerus gorhami

Corporaal, 1949 [Callimerus incertae sedis]

http://species-id.net/wiki/Callimerus_gorhami

Figs 81–83

gorhami Corporaal, 1949: 326 (*Callimerus*; type locality: “Tandjong Merah, Sumatra’s East Coast”); –Corporaal 1950: 90 (*Brachycallimerus*).

##### Type material examined.

**Holotype** of *Callimerus gorhami* Corporaal: “J. B. Corporaal, Sumatra’s O. K., Tandiong Merah, 18.XII.1917, 22m / Schenkling det. / S. Schenkling det. 1921: Callimerus pallidus Gorh. / Callimerus pallidus Gorh. [hw. by Schenkling] / J. B. Corporaal: Holotype: Brachycallimerus gorhami Corp., 1948 / ♀ / Brachycallimerus gorhami Corporaal, 1949, ZMAN type COLE. 1754.1” (ZMAN, ♀; Figs 81–83).

##### Note on Type material.

The original publication of *Callimerus gorhami* noted that only one specimen was examined, so the holotype was fixed in the original publication by monotypy.

##### Taxonomic position.

This species has a basal tooth on claw and metatibia with subapical projection on outer edge. Although it accords with the *latifrons* species-group in these two important characters, our exclusion of it from this species-group is based on the following characters: PL/PW >1 (ratio 1.1); EyD evidently larger than EyW (ratio 2.4); elytra apex with scales. Its nearest relative is still unclear.

## Discussion

Within these five species of the *latifrons* species-group, *Callimerus latesignatus*, *Callimerus pectoralis* and *Callimerus rusticus* seem to be most closely related. This assumption is supported by the apices of paramere convergent and EL/EW less than 2 (ratio 1.7–1.8); moreover, they have similar arrangement of elytral spots, with only different degrees of spots size and black pigmenting on ventral side. Furthermore, *Callimerus pectoralis* and *Callimerus rusticus* could beclosest to each other, because of their similarly shaped parameres and male sternite VIII. The distribution of *Callimerus pectoralis* and *Callimerus rusticus* shows a substitute pattern (Fig. 72), whereas *Callimerus latesignatus* is distributed to their northern border, and sympatric with *Callimerus pectoralis* in Yunnan, China.

**Figures 84–87.**
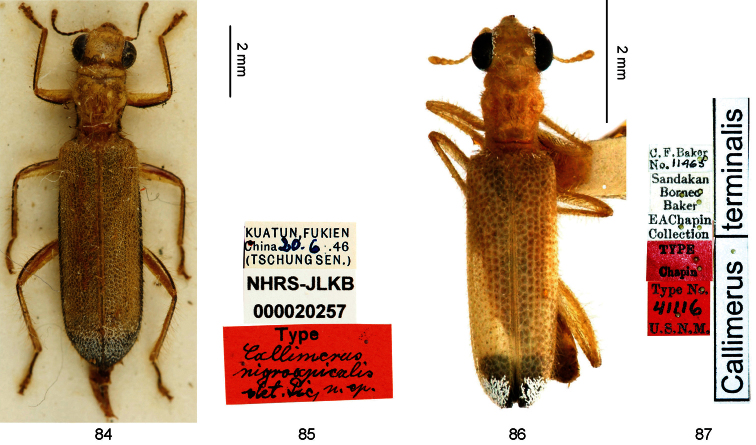
**84–85** Holotype of *Callimerus nigroapicalis* Pic, 1955 deposited in NHRS, photographed by J. Bergsten (**84** habitus **85** labels) **86–87** Holotype of *Callimerusterminalis* Chapin, 1919 deposited in USNM (**86** habitus **87** labels).

## Supplementary Material

XML Treatment for Callimerus latifrons

XML Treatment for Callimerus latifrons

XML Treatment for Callimerus latesignatus

XML Treatment for Callimerus pectoralis

XML Treatment for Callimerus rusticus

XML Treatment for Callimerus cacuminis

XML Treatment for Corynommadius trifasciatus

XML Treatment for Callimerus doesburgi

XML Treatment for Callimerus pallidus

XML Treatment for Callimerus gorhami
